# Health Tract No. 15

**Published:** 1865

**Authors:** 


### HEALTH TRACT No. 15.

### GROWING BEAUTIFUL

Persons may outgrow disease and become healthy by proper attention to the laws of their physical constitution. By moderate and daily exercise men may become active and strong in limb and muscle. But to grow beautiful, how? Age dims the lustre of the eye, and pales the roses on beauty's cheek; while crowfeet, and furrows, and wrinkles, and lost teeth, and gray hairs, and bald head, and tottering limbs, and limping most sadly mar the human form divine. But dim as the eye is, as pallid and sunken as may be the face of beauty, and frail and feeble that once strong, erect, and manly body, the immortal soul, just fledging its wings for its home in heaven, may look out through those faded windows as beautiful as the dew-drop of a summer's morning, as melting as the tears that glisten in affection's eye—by growing kindly, by cultivating sympathy with all human kind, by cherishing forbearance towards the follies and foibles of our race, and feeding, day by day, on that love to God and man which lifts us from the brute, and makes us akin to angels.

---

### WEAK EYES.

### WILLIS ON HALL.

(*From the Home Journal of Jan.* 28, 1860.)

A Good Hall.—A "very good haul," indeed, does he get, every month, who with a ***net*** dollar, ***takes*** the "Journal of Health," edited by Hall the Doctor! Of the pocket-wisdom most wanted, plain, pithy and pertinent, this little periodical, in our opinion, is the very purse. Now, what weak-eyed man or woman, for instance, will not be wiser for the following: "Many who are troubled with weak eyes, by avoiding the use of them in reading, sewing, and the like, ***until after breakfast***, will be able to use them with greater comfort for the remainder of the day, the reason being, that in the digestion of the food the blood is called in from all parts of the system, to a certain extent, to aid the stomach in that important process; besides, the food eaten gives general strength, imparts a stimulus to the whole man, and the eyes partake of their share."

---

The Door-bell Requiem.—To the belle men no longer adore, ***a door-bell*** tolls the requiem, (with its fewer-and-farther betweenities on New-Year's day,) or so seems to think Dr. Hall. Ah! the poetry there is—or might be—under the following statement of it in prose! "There are maiden ladies, who, some years ago, numbered their callers by dozens and scores, and ever hundreds; but for a few years past they have fallen off in geometrical progression, and now the diminution is really frightful. Formerly, when youth and beauty were theirs, the ***door-bell*** began to tingle as soon as the clock struck nine of the morning, with scarcely an intermission until it verged toward midnight. But now how great the change! Merry voices are heard outside, but they do not greet their ears; brisk footfalls sound on the pavement, but they do not stop at their doors, and a weary forenoon has almost passed away with only one or two visitors to break the disturbing monotony, former visions begin to assume more tangible shapes and the embodied idea stands out in high relief—***Passée!***"

